# The evaluation of risk prediction models in predicting outcomes after bariatric surgery: a prospective observational cohort pilot study

**DOI:** 10.1186/s13741-018-0088-5

**Published:** 2018-04-10

**Authors:** David Andrew Gilhooly, Michelle Cole, Suneetha Ramani Moonesinghe

**Affiliations:** 1 UCLH NIHR Surgical Outcomes Research Centre, Department of Anaesthesia and Perioperative Medicine, University College Hospital, London, NW1 2BU UK; 2 Department of Applied Health and Research, 1-19 Torrington Place, London, WE1C 7HB UK; 3 Bariatric Fellow, UCL Centre for Anaesthesia, University College London Hospital, London, NW1 2BU UK; 4 NIAA Health Services Research Centre, Churchill House, 35 Red Lion Square, London, WC1R 4SG UK

**Keywords:** Morbid obesity, Postoperative complications, Bariatric surgery, Risk assessment

## Abstract

**Background:**

As the prevalence of obesity is increasing, the number of patients requiring surgical intervention for obesity-related illness is also rising. The aim of this pilot study was to explore predictors of short-term morbidity and longer-term poor weight loss after bariatric surgery.

**Methods:**

This was a single-centre prospective observational cohort pilot study in patients undergoing bariatric surgery. We assessed the accuracy (discrimination and calibration) of two previously validated risk prediction models (the Physiological and Operative Severity Score for the enumeration of Morbidity and Mortality, POSSUM score, and the Obesity Surgical Mortality Risk Score, OS-MS) for postoperative outcome (postoperative morbidity defined using the Post Operative Morbidity Survey). We then tested the relationship between postoperative morbidity and longer-term weight loss outcome adjusting for known patient risk factors.

**Results:**

Complete data were collected on 197 patients who underwent surgery for obesity or obesity-related illnesses between March 2010 and September 2013. Results showed POSSUM and OS-MRS were less accurate at predicting Post Operative Morbidity Survey (POMS)-defined morbidity on day 3 than defining prolonged length of stay due to poor mobility and/or POMS-defined morbidity. Having fewer than 28 days alive and out of hospital within 30 days of surgery was predictive of poor weight loss at 1 year, independent of POSSUM-defined risk (odds ratio 2.6; 95% confidence interval 1.28–5.24).

**Conclusions:**

POSSUM may be used to predict patients who will have prolonged postoperative LOS after bariatric surgery due to morbidity or poor mobility. However, independent of POSSUM score, having less than 28 days alive and out of hospital predicted poor weight loss outcome at 1 year. This adds to the literature that postoperative complications are independently associated with poor longer-term surgical outcomes.

## Background

Obesity is one of the twenty-first century’s pre-eminent public health problems. The World Health Organization (WHO) estimates that there are 2.3 billion overweight people globally, of which 700 million are obese (W.H.O 2018). A report by the UN Food and Agriculture Organization in 2013 showed that 24.9% of people in the United Kingdom (UK) were considered obese and that the UK was at the top of Europe’s obesity league table (The State of Food and Agriculture 2013). In the United States of America (USA), the prevalence is even higher with data showing that more than one in three adults are considered obese (Flegal et al. 2012).

High levels of obesity put significant burden on health services as a result of associated comorbidities. It has been estimated that the direct cost to the NHS of treating overweight and obese people was £4.2 billion in 2007 (Butland et al. 2007). The UK’s National Bariatric Register shows that 53.9% of men and 41.4% of women had four or more obesity-related diseases at the time of primary surgery (Welbourn et al. 2014). However, significant improvement, if not resolution, of comorbidities can occur within 2 years of bariatric surgery (Welbourn et al. 2014; Arterburn and Courcoulas 2014; Colquitt et al. 2014) with long-term cost savings due to treatment of not just obesity, but obesity-related illnesses ((UK) NCGC 2014).

The UK second National Bariatric Register report has shown that 16,956 primary bariatric surgical procedures were performed between 2001 and 2013, 95% of which were performed laparoscopically. In this cohort, surgical complication rates were 2.9% and observed in-hospital mortality 0.07% (Welbourn et al. 2014). With such low mortality rates, monitoring morbidity or complications may provide clinicians and patients with more useful information on quality and variation in standards of care and provide a greater opportunity for performance improvement.

Although weight loss is not considered to be the most important outcome of bariatric surgery (rather, the aim is to support resolution of obesity-related illnesses), it is nevertheless an important proxy of surgical effectiveness (Welbourn et al. 2014). Factors that have been found to influence various outcomes include higher body mass index (BMI), age, increase in number of comorbidities and American Society of Anesthesiologists’-Physical Status (ASA-PS) (Colquitt et al. 2014; Abraham et al. 2015). Of note, postoperative complications can vary in incidence depending on the definition of complication being used.

Finding an accurate risk stratification tool is important so that patients at higher risk of postoperative morbidity can be identified and their perioperative pathway optimised to drive better surgical outcomes. Studies have previously looked at the OS-MRS as a tool for prediction of perioperative outcome with variable results (Coblijn et al. 2016; Lorente et al. 2014), but this scoring system was designed and validated as a predictor of mortality and not morbidity (DeMaria et al. 2007). The Physiology and Operative Severity Score for the enUmeration of Morbidity and Mortality has been previously suggested as the most well-validated risk stratification model for predicting morbidity in heterogeneous patient populations (Moonesinghe et al. 2013), but previous research in bariatric surgery found it overestimated postoperative morbidity (Charalampakis et al. 2014).

The aim of this study was to evaluate two previously developed and validated scores, the POSSUM and OS-MRS scores, for the prediction of postoperative morbidity and longer-term weight loss at 1 year. In addition, we also evaluated independent predictors for poor weight loss using multivariable analysis.

## Methods

This single-centre observational cohort pilot study was approved by the University College London Hospitals NHS Foundation Trust’s (UCLH) Research and Development office as a service evaluation. Between 01 March 2010 and 30 September 2013, data were collected prospectively on consecutive adult (> 18 years) patients undergoing bariatric surgery which included sleeve gastrectomy and laparoscopic Roux-en-Y gastric by-pass (RYGB) procedures at University College Hospital, a London teaching hospital.

### Patient pathway

Patients initially attended a combined bariatric outpatient clinic where they were seen by the dietician, bariatric nurse specialist, bariatric surgeon and endocrinologist. Initial weights of patients were documented. Cases were then reviewed at a multidisciplinary meeting, and suitable cases were listed for surgery. After discharge from hospital, patients were followed up by the surgical bariatric team for outcomes and complications (Grocott et al. 2007) at regular intervals of 6 weeks and 3, 6, 12 and 18 to 24 months. Outpatient clinic weight measurements were routinely taken during follow-up appointments, and incidence of all complications were determined by case note review.

### Predictor variables

Data were collected by a trained research team working within the UCLH NIHR Surgical Outcomes Research Centre (SOuRCe). Demographics collected on all patients included age, weight, BMI, ethnicity, gender, attendance to pre-assessment, comorbidities, American Society of Anesthesiologists’ Physical Status, grade of attending surgeon and anaesthetist, operation performed, postoperative care ward and necessary investigations to calculate POSSUM and OS-MRS scores. The POSSUM score is calculated using a combination of 12 physiological and 6 operative data variables for each patient to calculate percentage risk. Originally developed in 1991 by Copeland et al. (Copeland et al. 1991), it has been evaluated widely, including in orthopaedic, vascular, head and neck and colorectal surgeries (Mohamed et al. 2002; Prytherch et al. 2001; Myers 1993; Griffiths et al. 2002; Tekkis et al. 2000). The OS-MRS uses a binary point scoring system based on five variables to stratify patients into three main groups (DeMaria et al. 2007). It is currently the most commonly used risk stratification tool for bariatric surgery (Daniel Guerron and Portenier 2016) and has been shown to be a useful tool for morbidity prediction as well (Lorente et al. 2014; Pinho et al. 2015).

### Outcome measures

The primary outcome was poor weight loss, defined as < 50% percentage of excess body weight loss (EBWL) at 1 year postoperatively. Secondary outcomes included inpatient postsurgical morbidity, measured using the Post Operative Morbidity Survey (POMS) on day 3 after surgery (Grocott et al. 2007), and length of hospital stay. The POMS has been previously validated as a measure of morbidity which necessitates hospital admission (Grocott et al. 2007; Davies et al. 2013; Goodman et al. 2015). Day 3 POMS-defined morbidity was selected as the primary outcome measure as the national UK average postoperative stay has been reported as 2.7 days (Welbourn et al. 2014), and therefore, we hypothesised that day 3 morbidity would represent a departure from the usual postoperative pathway. If a patient was already discharged from hospital by day 3, the patient was recorded as being morbidity free, as previously described (Grocott et al. 2007). In order to capture the impact of serious adverse events occurring after the initial discharge from hospital, such as short-term mortality and hospital readmissions, we also report the composite endpoint of days alive and hospital free at 30 days post-surgery—this has been colloquially termed ‘happy days’ (Moonesinghe et al. 2017).

### Statistical analysis

Continuous variables are presented as mean (SD) when normally distributed and median (range) when not (normality was assessed using the Stata ‘sktest’ for skewness and kurtosis in large sample sizes). Categorical variables are presented as *n* (%). Both POMS-defined morbidity according to the originally defined 9 physiological domains and prolonged length of stay due to failure to return to preoperative level of mobility were recorded and analysed separately. We tested the predictive accuracy (discrimination and calibration) of the ASA-PS score, (Saklad 1941) OS-MRS and POSSUM morbidity equation for predicting prolonged length of stay with morbidity defined using the POMS. Discrimination was assessed by analysing the area under the receiver-operator-characteristic curve (AUROC) and calibration measured, using the Hosmer-Lemeshow (HL) chi-squared statistic. A priori, we determined that AUROC > 0.9 would indicate good discrimination, 0.6–0.9 would indicate moderate and < 0.6 would indicate poor performance (Swets 1988). Calibration gives an estimation of how good the model is at predicting the probability of the event occurring across the full range of outcomes in that population. We assessed the calibration of the POSSUM score, using the Hosmer-Lemeshow chi-squared statistic, with significance set at *p* > 0.05.

The morbidity prediction model with the highest discrimination was then used to adjust for patient risk factors in an analysis, which tested the independent relationship between postoperative morbidity and poor longer-term outcome (defined by EBWL less than 50% at 1 year follow-up).

## Results (Fig. 1)

### Baseline patient characteristics

Two hundred and thirty-one patients underwent bariatric surgery during the study period and had demographics collected by the SOuRCe team. This was then collated with the surgical postoperative database. One hundred and ninety-seven patients were included in the analyses. Demographics are shown in Table 1. All 197 patients had their weight recorded at 1 year.

**Fig. 1:**
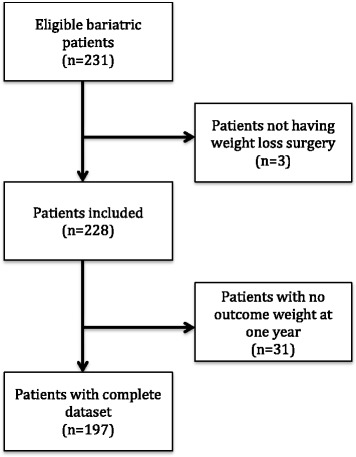
Flow diagram for cases included and excluded from analysis

**Table 1:** Characteristics of patients with complete data collected

Characteristics	Complete data *n* = 197	No outcome weight *n* = 34	*p*
Mean age (SD)	45 years (12.8)	44.34 (11.6)	*p* = 0.63
Sex			
Male	21.3%	22.9%	*p* = 1.0
Female	79.7%	77.1%	
Ethnicity			
White British	68.5%	68.6%	*p* = 1.0
Other	31.5%	31.4%	
Attended pre-assessment	96.5%	91.4%	*p* = 0.072
Smoker			*p* = 0.199
Current	12.7%	11.4%	
Ex-smoker	25.4%	40%	
Non-smoker	61.9%	48.6%	
Alcohol			*p* = 0.251
Current drinker	52.2%	50%	
Non-drinker	47.8%	50%	
ASA-PS			*p* = 0.892
ASA 1	13.7%	8.5%	
ASA 2	67%	68.6%	
ASA 3	19.3%	20.0%	
ASA 4	0%	2.8%	
Diabetic			*p* = 0.73
Insulin controlled	3.6%	5.7%	
Tablet controlled	24.9%	17.1%	
Diet controlled	2%	2.9%	
Non-diabetic	69.5%	74.3%	
Other comorbidities			*p* < 0.001
Ischaemic heart disease	3%	0%	
Liver disease	1.5%	0%	
OS-MRS mean (SD)	1.09 (0.77)	1.17 (0.98)	*p* = 0.564
POSSUM physiology mean (SD)	14.48 (2.63)	15.6 (2.24)	*p* = 0.013
POSSUM operative mean (SD)	9.11 (0.64)	8.34 (0.94)	*p* < 0.001 95% CI (0.52–1.02)
Postoperative destination			*p* = 0.42
Intensive care	0.5%	0%	
Post anaesthetic care unit (PACU)	36.6%	45.7%	
Ward	62.9%	54.3%	

POSSUM scores were calculated for all patients and divided into physiological, operative and total POSSUM scores. The median POSSUM physiology score was 14 (IQR 13–15), the median POSSUM operative score was 9 (IQR 9–9) and total POSSUM median score was 22 (IQR 22–24).

The most common procedure was a laparoscopic sleeve gastrectomy, (59.9%), followed by laparoscopic Roux-en-Y gastric by-pass procedures (38%). Of the remaining procedures, one was converted from a sleeve gastrectomy to a RYGB, one had a cholecystectomy with the procedure, one had a hiatus hernia repair and one was converted to an open procedure.

### Postoperative outcomes

Postoperatively, 124 patients were admitted to the general ward (62.9%); 72 were admitted to post anaesthetic care unit (PACU) (36.5%), a high dependency unit designated for post surgical patients; and one was admitted to ICU. The median length of stay (LOS) was 2 days postoperatively (IQR range 2–3 days).

There were two inpatient hospital deaths (mortality = 0.85%); all patients who were discharged went home to their usual residence. Seventeen patients (8.6%) were readmitted within 30 days of hospital discharge, of whom seven had a readmission stay longer than 3 days. The date of readmission varied between 1 and 30 days post-discharge (median 4, IQR 2–19.5). The most common reason for re-admission was abdominal pain (8 patients—47% of readmissions or 4% of the total cohort), and 3 patients (17.6% of readmissions, 1.5% of the total cohort) had an anastomotic leak.

The mean %EBWL at 12 months was 56.86%, (SD 19.9%). Seventy-nine patients (40.1%) had an EBWL less than 50% at 1-year follow-up.

One hundred thirty-eight patients were discharged by day 3; thus, 59 patients (30%) remained in hospital and had day 3 POMS data collected. Table 2 shows the POMS-defined morbidity on days 3, 5, 7, 14 and 21.

**Table 2:** Number (and percentage) of patients with POMS-defined morbidity for each collection day and the total inpatient number on that day (denominator 197 patients)

	POD 3 (%)	POD 5 (%)	POD 7 (%)	POD 14 (%)	POD 21 (%)
Number of patients in hospital	59 (30.0)	9 (4.6)	5 (2.5)	2 (1.0)	1 (0.5)
Number of patients POMS + (excluding mobility)	51 (25.9)	7 (3.5)	5 (2.5)	2 (1.0)	1 (0.5)
Respiratory	7 (3.5)	5 (2.5)	2 (1.0)	0	0
Infection	5 (2.5)	4 (2.0)	4 (2.0)	0	0
Renal	7 (3.5)	3 (1.5)	2 (1.0)	0	0
Gastrointestinal	48 (24.4)	3 (1.5)	3 (1.5)	1 (0.5)	0
Cardiovascular	3 (1.5)	0	0	0	1 (0.5)
Wound	3 (1.5)	3 (1.5)	1 (0.5)	0	0
Haematological	4 (2.0)	0	0	0	0
Neurological	0	0	0	0	0
Pain	9 (4.6)	2 (1.0)	1 (0.5)	1 (0.5)	0
Mobility	59 (29.9)	3 (1.5)	0	0	0

Note that mobility is not part of the original POMS domains but is widely used to help define reasons for continued hospital stay other than morbidity

*POD* postoperative day

### Risk prediction according to POSSUM and OS-MRS

Both POSSUM and OS-MRS were less accurate in predicting POMS-defined morbidity on day 3 than predicting the composite of prolonged length of stay due to poor mobility and/or POMS-defined morbidity. Neither of these tools reached moderate predictive accuracy for predicting POMS-defined morbidity on day 3, but both were moderately accurate for predicting inpatient stay ≥ 3 days due to morbidity or poor mobility. In order to be able to evaluate the predictive accuracy of ‘happy days’ for poor weight loss at 1 year, we converted this into a binary variable for the purpose of estimating AUROC by assigning a poor outcome to patients who had less than 28 days alive and out of hospital (as the median length of stay of the primary admission was 2 days). In multivariable analysis, considering POSSUM-defined risk, age, and whether or not the patient had < 28 days alive and out of hospital by 30 days post-discharge, the only independent predictor of a poor EBWL outcome at 1 year was having less than 28 days alive and out of hospital (odds ratio (OR) 2.6; 95% confidence interval (95% CI 1.28–5.24)) followed by age (OR 1.03; 95% CI 1.00–1.06) (Table 3).

**Table 3:** Discrimination and calibration of risk prediction tools for morbidity outcomes

	AUROC POMS-defined morbidity on D3: AUROC (95% CI; standard error)	HL statistic (*p* value)	AUROC POMS-defined morbidity or failure to return to preoperative mobility on D3	HL statistic (*p* value)	EBWL < 50% at 1 year	HL statistic (*p* value)	AUROC: < 28 days alive and out of hospital	HL statistic (*p* value)
POSSUM	0.60 (0.50–0.69; 0.04)	1.85 (0.87)	0.63 (0.55–0.72)	1.36 (0.93)	0.60 (0.52–0.69; 0.04)	11.64 (0.04)	0.66 (0.58–0.74; 0.04)	19.74 (0.47)
OS-MRS	0.62 (0.53–0.70; 0.05)	NA	0.63 (0.55–0.71)	NA	0.59 (0.50–0.67; 0.04)	NA	0.62 (0.55 0.70; 0.04)	NA

Note: No HL statistics calculated for the OS-MRS as this is an ordinal scale rather than providing a percentage prediction of the outcome

*POSSUM* Physiology and Operative Severity Score for the enUmeration of Morbidity and Mortality, *OS-MRS* Obesity Surgery Mortality risk Score, *AUROC* area under the receiver-operator-characteristic curve, *HL statistic* Hosmer-Lemeshow chi-squared statistic, *POMS* Post Operative Morbidity Survey, *EBWL* excess body weight loss

## Discussion

In this study, we report the accuracy of POSSUM and OS-MRS in predicting postoperative morbidity in patients undergoing bariatric surgery. Both were shown to be poor predictors of POMS-defined morbidity on day 3 but showed moderate accuracy at predicting inpatient LOS ≥ 3 days due to morbidity or poor mobility. The average LOS of bariatric patients postoperatively has been reported nationally as 2.7 days (Welbourn et al. 2014), so these tools may be useful in predicting patients who are at risk of increased resource utilization. In this analysis, the strongest independent predictor of poor weight loss at 1 year (< 50% EBLW) was the failure to have > 28 days alive and out of hospital within the first 30 days of surgery—a composite endpoint of death, length of hospital stay and readmission to hospital.

It is important to risk stratify patients for bariatric surgery in order to facilitate optimal allocation of resources. There is no previously validated risk tool for sleeve gastrectomies and RYGB procedures, which comprise the majority of bariatric procedures. Three US studies have evaluated morbidity risk prediction models for bariatric surgery, all of which used the American College of Surgeons National Surgical Quality Improvement Program (ACS-NSQIP) as their source of data. Turner et al. reviewed data from 2005 to 2008 and derived a normogram based on four factors (age, BMI, albumin and functional status); the C-statistic (which is equal to the AUROC) in the validation cohort was 0.629 (Turner et al. 2011). Although this study analysed over 32,000 bariatric procedures, these did not include sleeve gastrectomies (Gupta et al. 2011). Gupta et al. derived a risk prediction model based on six factors using the 2007 ACS-NSQIP dataset: the variables included recent myocardial infarction/angina, functional status, stroke, bleeding disorder, hypertension, BMI and type of bariatric surgery. The C-statistic for this model was 0.66 (Gupta et al. 2011) with almost a third of the cases were gastric band procedures, which are less commonly performed now. More recently, Aminian et al. used the 2012 ACS-NSQIP dataset to develop a model for laparoscopic sleeve resections comprising seven variables (congestive heart failure, chronic steroid use, male sex, diabetes, preoperative serum bilirubin, BMI and preoperative haematocrit). This model appeared to be the most promising, with a C-statistic of 0.682 (Aminian et al. 2015).

Our analyses found that POSSUM and OS-MRS were moderately accurate for predicting stay ≥ 3 days due to morbidity and poor mobility, with AUROC 0.63 for both. They were less accurate in predicting POMS-defined morbidity on day 3. Although they have not been shown to be a significant predictor of EBWL at 1 year, they do predict increased length of stay in hospital. Using one of these systems in the preoperative assessment clinic may support clinicians in identifying patients who may benefit from admission to the PACU (Daniel Guerron and Portenier 2016) or more intensive after-care pathways, including physiotherapy and occupational therapy. Preoperative optimization of these patients, or pre-habilitation, may be also of benefit to this demographic as it can improve physical fitness, which can help to improve outcomes (West et al. 2015; Bond et al. 2015).

Independent of the patient’s preoperative health status, a complicated postoperative course predicted poor weight loss at 1 year. This observation adds to the body of evidence that postoperative morbidity may have lasting impact on patient outcomes, which outlast the resolution of the overt complication, and which makes the prevention of postoperative morbidity an important goal of quality improvement (Moonesinghe et al. 2014; Khuri et al. 2005). Weight loss after bariatric surgery is not a certainty and requires the patient to be supported by a multidisciplinary team to achieve this. Patients require regular follow-up in the first 2 years post-surgery to ensure lifestyle changes occur, continued nutritional support and identify any maladaptive eating disorders (Metcalf et al. 2005). Postoperative surgical complications and prolonged recovery also have been shown to have an adverse effect on patient psychology (Pinto et al. 2016). Together, these factors may contribute to a poor outcome through lack of engagement and inability to access the necessary postoperative support as a result of their morbidity. In a patient population already at risk of depression (Carey et al. 2014), the added stress of complications may compound this risk and added to probable immobility as a result of postoperative complications, may result in a more sedentary lifestyle.

We also found an association between age and EBWL at 1 year, with a 3% increase in the risk of not achieving target weight loss, per year of advancing age. Previous analyses from large US cohorts have found conflicting evidence on this. A prospective observational study of 4776 patients evaluating 30-day outcomes (Flum et al. 2009) found no association between age and morbidity or mortality. A subsequent retrospective cohort analysis of 48,378 patients who underwent bariatric surgery in the 2005–2009 American College of Surgeons National Surgical Quality Improvement program (ACS-NSQIP) (Dorman et al. 2012) found older age was associated with prolonged LOS but not major adverse events. However, two more recent publications from the ACS-NSQIP of 44,408 (Khan et al. 2013) and 20,308 (Sanni et al. 2014) patients respectively showed an association between increasing age and morbidity and mortality. The latter study found that the odds of postoperative complications increased by 2% with each additional year of age. An analysis of 8945 patients from the Bariatric Outcome Longitudinal Database found that women and younger patients had significantly more weight loss (Van De Laar 2014).

Finally, comparing the POSSUM and OS-MRS, both show similar low accuracy in predicting postoperative morbidity at day 3 and moderate accuracy for predicting prolonged LOS due to morbidity or poor mobility, or ‘happy days’. As there is no tool for prediction of morbidity related to current bariatric surgery practices, either of these could act as a tool. This paper also highlights the need for a larger study to define a risk prediction tool for morbidity in bariatric surgery.

### Clinical implications

From this study, we can hypothesise that patients with a higher POSSUM score and older patients may benefit from more intensive perioperative care. Candidate interventions might include those which have been found to be associated with improved outcomes in other settings, such as goal-directed therapy, (Grocott et al. 2013; Hamilton et al. 2011), enhanced recovery (Grocott et al. 2012; Barreca et al. 2015) or admitting these patients to a critical care setting after surgery (Alfa Wali et al. 2014). However, randomised trials of these interventions in bariatric surgical patients are required to answer these questions.

### Limitations

This study was undertaken at a single centre, and this may affect the generalizability of our findings. The psychological status of the patient plays an important role in the final surgical outcome after bariatric surgery; in our analyses, this factor was not taken into account for two reasons: not all patients had a psychological assessment prior to surgery and results of such an assessment can be difficult to describe quantitatively.

In our study, the postoperative morbidity rates appear much higher than those quoted from our national register (33 vs 2.9%). It has been shown that morbidity can vary widely between different studies, depending, at least in part, on how you classify complications (Colquitt et al. 2014). The comparatively high morbidity rate in our study is likely to be because the POMS include relatively minor morbidities; an alternative definition might be to describe this as ‘absence of full recovery’. The most common type of morbidity on D3 was gastrointestinal, and in most cases, this was due to nausea, vomiting or abdominal distension—which would not commonly appear as a ‘complication’ in other classification systems.

## Conclusion

As the demand for surgery to treat the obesity epidemic increases, it will become increasingly important to risk stratify patients in order to effectively plan perioperative care. The mortality associated with surgery is very low but there is a need to reduce postoperative morbidity, which can have an effect on hospital resource utilization and is associated with reduced postoperative weight loss. Although the POSSUM and OS-MRS scores have been shown in this study only to be moderately effective at predicting outcome for both sleeve gastrectomies and RYGB procedures, they are equivalent to previously published analyses of other models in large US cohorts (Turner et al. 2011; Gupta et al. 2011; Aminian et al. 2015). Further, none of these US models have been validated on populations of patients undergoing the two most common bariatric procedures undertaken currently. Validation of our findings in multi-centre cohorts would be of value.

## Abbreviations

ACS-NSQIP: American College of Surgeons National Surgical Quality Improvement Program
ASA-PS: American Society of Anesthesiologists’-Physical Status
AUROC: Area under the receiver-operator-characteristic curve
BMI: Body mass index
CI: Confidence interval
EBWL: Excess body weight loss
HL: Hosmer-Lemeshow
IQR: Inter quartile range
LOS: Length of stay
NA: Not applicable
NHS: National Health Service
NIHR: National Institute for Health Research
OR: Odds ratio
OS-MRS: Obesity Surgical Mortality Risk Score
POMS: Post Operative Morbidity Survey
POSSUM: Physiology and Operative Severity Score for the enUmeration of Morbidity and Mortality
RYGB: Roux-en-Y gastric by-pass
SD: Standard deviation
SOuRCe: Surgical Outcomes Research Centre
UCLH: University College London Hospitals
UN: United Nations
WHO: World Health Organization
